# Single-Photon Single-Particle Tracking

**DOI:** 10.1101/2025.01.10.632389

**Published:** 2025-10-15

**Authors:** Lance W.Q. Xu, Nathan Ronceray, Marianna Fanouria Mitsioni, Titouan Brossy, David Štastný, Radek Šachl, Martin Hof, Aleksandra Radenovic, Steve Pressé

**Affiliations:** 1 Center for Biological Physics, Arizona State University, Tempe, AZ, USA; 2 Department of Physics, Arizona State University, Tempe, AZ, USA; 3 Institute of Bioengineering, School of Engineering, EPFL, Lausanne, Switzerland; 4 NCCR Bio-Inspired Materials, EPFL, Lausanne, Switzerland; 5 J. Heyrovský Institute of Physical Chemistry, Dolejškova 2155/3, CZ-18223 Prague 8, Czech Republic; 6 Faculty of Science, Charles University, Prague, Czech Republic; 7 School of Molecular Sciences, Arizona State University, Tempe, AZ, USA

## Abstract

Mobile biological particles, ranging from biomolecules to viral capsids, often diffuse faster than 1*μ*m^2^/s, resulting in severe motion blur in conventional millisecond-scale imaging. While shorter exposures may help provide the data needed to capture faster dynamics, quantization of signal intensity per pixel at such exposures eventually interferes with our ability to track. In the extreme case of binary (1-bit-per-pixel) output—where going from 8-bit conventional grayscale imaging to 1-bit directly corresponds to a 255-fold faster acquisition rate—no existing tracking methods can be used, as these methods fundamentally rely on intensity-based localization, which does not leverage the binary output. For this reason, we introduce single-photon single-particle tracking (SP^2^T), a framework that bypasses localization and linking by estimating particle numbers and trajectories directly by jointly considering 1-bit image stacks. While cameras capable of microsecond-scale exposures, typically based on scientific CMOS (sCMOS) sensors or single-photon detectors (SPDs), are increasingly central to this effort, in this work, we focus on single-photon avalanche diode (SPAD). Single-photon detector (SPD) arrays offer microsecond exposures over large fields of view (512×512 pixels). SP^2^T accounts for detector-specific artifacts such as hot and cold pixels and is validated with programmed fluorescent bead trajectories and biological systems (aerolysin and ganglioside). These experiments, in addition to simulations, reveal that analysis performed with longer exposures can bias diffusion coefficient estimates (up to 70% for particles with diffusion coefficients of 5*μ*m^2^/s) and distort jump-distance distributions, underscoring the need for photon-by-photon tracking in fast-diffusion regimes. Moreover, SP^2^T delivers substantial computational gains—achieving more than a 50-fold GPU speedup over CPU-based likelihood tracking methods that assume continuous intensity, when compared on datasets with the same frame size and number of frames. Together, these advances establish SP^2^T as a robust, data-efficient solution for unbiased particle tracking with millisecond-to-microsecond temporal resolution.

## Introduction

Advances in fluorophore photophysics have allowed us to surpass the diffraction limit of light, enabling fluorescent localization of molecular positions with remarkable precision, down to approximately 70nm^1–14^. More recent techniques have pushed this precision even further, with localization down to 6nm achieved through excitation beam modulation^15^, with subsequent *in vitro* techniques reaching sub-nanometer precision^16^. With these advancements, we can now directly observe several biological structures^17^, nanoparticle endocytosis, and targeted drug delivery to cancer sites^18,19^, as well as viral particle activity within living systems^20,21^.

**Figure 1:**
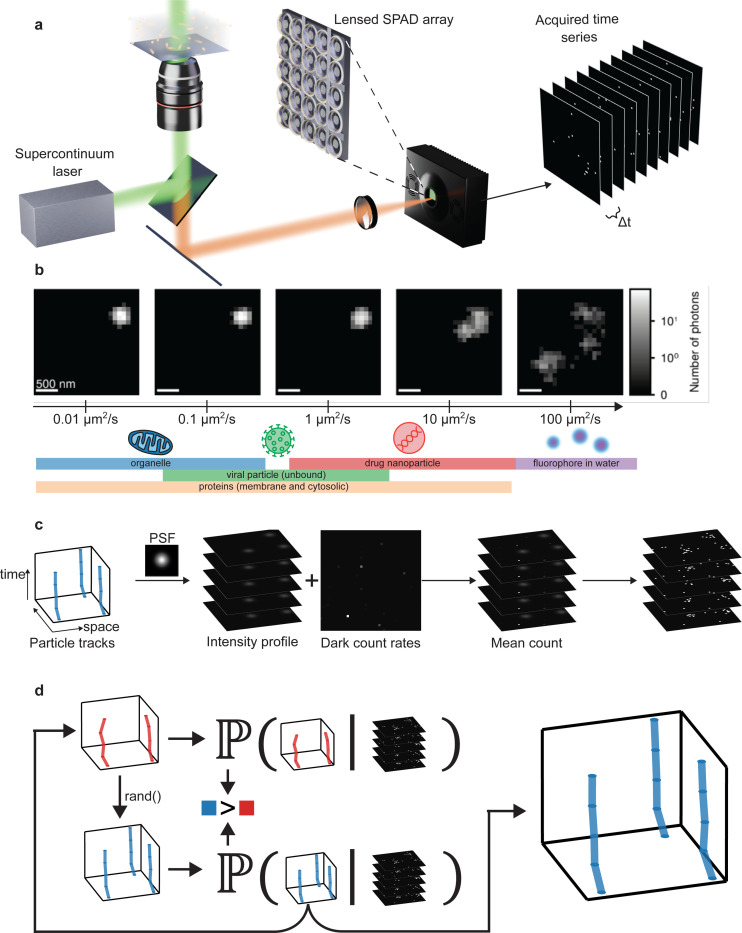
**a**, Schematic of the experimental setup. A supercontinuum pulsed laser source provides illumination, with the option to switch between epi-illumination and total internal reflection fluorescence modes. Image acquisition is performed using the SwissSPAD^2^ camera at frame rates up to 97.7kHz. Only a subset of the full 512×512 pixel (FOV) is shown for illustrative purposes. **b**, Typical diffusion coefficient ranges for different biological systems^47^. Motion blur begins to appear at diffusion coefficients as low as 1*μ*m^2^/s with an exposure time of 10ms, potentially causing a single particle to be misinterpreted as multiple distinct objects, as shown in Fig. S.2 and as becomes apparent as we move beyond 1*μ*m^2^/s. **c**, As a physics-inspired model, SP^2^T considers the process by which data is generated from the fluorescently labeled particles, including the system’s point spread function (PSF) and the dark count rate of each pixel. **d**, A Monte Carlo algorithm to sample candidate particle tracks from the posterior probability distribution. Based on the current track sample (red), we propose a new particle with its associated track (blue) and compute corresponding posterior probabilities. Favoring higher probability, we stochastically accept or reject this new track and update the current sample. In this way, we propose particle numbers and their associated tracks consistent with the entirety of the FOV over all frames and all of the optics systematically integrated into the probability.

Despite remarkable advances in spatial resolution, achieving high temporal resolution remains a significant challenge. Motion blur arises readily at typical exposure times (≈10ms), with visible artifacts even at diffusion coefficients as low as 1 *μ*m^2^/s (Fig. 1b). This limitation becomes critical when imaging faster diffusing molecules: SARS-CoV-2 virions diffuse at rates exceeding 5 *μ*m^2^/s^22^, and cytoplasmic proteins can reach 10 *μ*m^2^/s^23^. Moreover, motion during exposure not only degrades localization precision but also introduces ambiguity in identifying individual molecules, especially when blurred signals overlap with nearby emitters. Recent work with MINFLUX^15^, a real-time tracking method, shows that slow acquisition rates may even lead to systematic underestimation of diffusion coefficients^24^, underscoring that acquisition speed remains a fundamental constraint across modalities. These challenges are further exacerbated by the diffraction-limited point spread function (PSF), which causes even small biomolecules to appear as extended features (250nm)^25^.

Stroboscopic illumination partially mitigates motion blur by narrowing the effective exposure window^26–28^. However, by sampling intermittently, it risks missing transient events between flashes, introducing tracking errors such as trajectory mislinking (Fig. S.1). As a result, while effective for reducing blur, stroboscopic imaging provides only a partial solution to the problem of temporal resolution.

Truly overcoming these limitations requires continuous high-speed acquisition. Cameras capable of microsecond-scale exposures, typically based on scientific CMOS (sCMOS) sensors or single-photon detectors (SPDs), are increasingly central to this effort. SPDs encompass several device classes, including photomultiplier tubes (PMTs), single-photon avalanche diodes (SPADs), superconducting nanowire single-photon detectors (SNSPDs), and transition-edge sensors (TES)^29^. These detectors are increasingly widely adopted in biological imaging due to their absence of readout noise and their ability to perform parallel single-photon counting and time-resolved measurements^30–37^.

In this work, we focus on single-photon avalanche diode (SPAD) arrays as a specific iteration of detector architectures generating rapid single-photon-per-pixel output. More specifically, we focus on the SPAD512^2^ from Pi Imaging. This sensor combines a large field of view (512×512 pixels) with a high data acquisition rate of 97.7kHz, making it well-suited for high-speed imaging applications. Designed to operate reliably at or slightly below room temperature, the SPAD512^2^ features a compact form factor, low operating voltage, and robust performance across a range of conditions^38^. SPAD arrays have already demonstrated utility in diverse domains, including fluorescence microscopy^30–32,34,35^, time-resolved spectroscopy^39–41^, and nuclear medicine^41^. Beyond their exceptional temporal resolution, SPAD technology also enables fluorescence lifetime imaging, providing detailed insights into molecular species and their local environments.

Despite the rapid data acquisition rates of SPAD arrays and other devices capable of raw 1-bit (single-photon) output, no existing method can directly extract dynamical information from such data. The central challenge is that tracking requires localizing light-emitting particles in each frame, yet most localization algorithms depend on sufficient detected photons (*i.e.*, pixel intensities) to achieve high accuracy^42^. This requirement fundamentally conflicts with the discrete, single-photon nature of SPD data. Consequently, recent studies with SPDs, including SPAD arrays, still rely on binning consecutive 1-bit frames for analysis^37,43^, a process that sacrifices the exceptional temporal resolution these detectors can provide (Fig. 1). While binning enables particle localization with conventional tracking tools, it does not add information; rather, it averages away the intrinsic temporal detail of the dataset^44–46^. This highlights the need for methods that bypass binning and instead leverage the full information content of raw 1-bit frames.

Here, we introduce single-photon single-particle tracking (SP^2^T), a framework designed to enable direct particle tracking from 1-bit frames. SP^2^T fundamentally departs from the conventional localization paradigm by simultaneously proposing the number of particles and their trajectories in a manner consistent across the entirety of the 1-bit-per-pixel image stack.

Unlike the analysis for integrative detectors, an accurate SPAD array algorithm requires careful calibration of each pixel. For instance, hot pixels can be confounded for additional particles by consistently registering false-positive detections. In contrast, cold pixels may create areas that appear to repel particles, thereby generating trajectories avoiding those regions. Thus, as we will see in SP^2^T, we account for realistic complications in SPAD array detectors, including hot and cold pixels.

## Results

To validate whether SP^2^T can reliably track particles using 1-bit frames, we begin with controlled fluorescent bead experiments, where beads are fixed to a piezoelectric (piezo) stage programmed to follow a known trajectory. This setup allows for direct comparison between the programmed ground truth and the tracks inferred by SP^2^T. Next, we apply SP^2^T to a biologically relevant aerolysin dataset, in which monomers are tracked in bilayers. Unlike fluorescent beads, aerolysin molecules are labeled with Cy3B, making them approximately half as bright and thereby testing the robustness of tracking under lower photon counts. We then demonstrate the critical importance of analyzing 1-bit frames acquired at short exposure times using a dataset of diffusing ganglioside GM1 molecules, which allows accurate estimation of diffusion coefficients and correct inference of the underlying diffusive models. To highlight multiplexing capability, we further analyze the aerolysin dataset at full FOV, showing that SP^2^T can simultaneously track many emitters across large imaging regions. Finally, we assess robustness using synthetic datasets spanning a wide range of conditions, including emitter separation, diffusion coefficients, emission brightness, and dark count rates, demonstrating the adaptability of SP^2^T across diverse experimental scenarios.

As SP^2^T is based on a probabilistic framework within the Bayesian paradigm^48^, it predicts full distributions over particle numbers and associated tracks obtained by drawing samples from the posterior probability distribution, not just point estimates over particle numbers and their most probable tracks. However, to plot our results in an interpretable manner, we typically plot point estimates obtained as the *maximum a posteriori* (MAP) sample representing the particle numbers and their associated tracks, maximizing the posterior. We generate multiple posterior samples to quantify uncertainty, typically around 500 for each track.

### Fluorescent beads moving with a piezo stage

The first control experiments we present involve fluorescent beads fixed to a piezo stage following a pre-programmed trajectory, assuring that the ground truth is known. This trajectory is designed by holding the stage stationary for a constant dwell time (40ms), then moving by a predetermined step size (200nm) in a randomly chosen direction: left, right, up, or down.

This setup allows us to: 1) directly validate our tracking results against the known programmed trajectories; 2) assess the consistency of the inferred tracks across multiple beads, thereby confirming the robustness of our framework; and 3) benchmark our results against those obtained using TrackMate^49^ on binned frames. These comparisons demonstrate that SP^2^T resolves particle tracks below the diffraction limit while achieving temporal resolution at least an order of magnitude faster.

**Figure 2:**
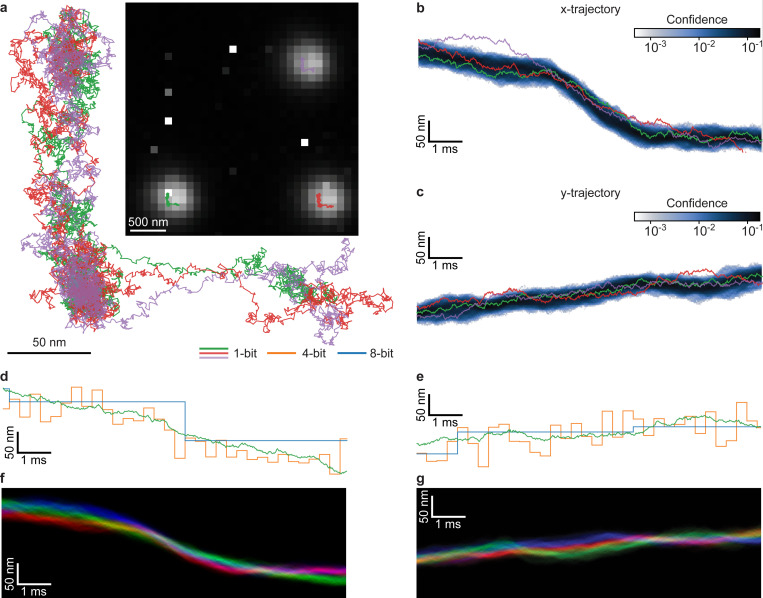
Results of fluorescent beads moving with a piezo stage with 40ms dwell time and 200nm step size. **a**, An example frame generated by summing all 5,750 45×45 1-bit frames with 10*μ*s exposure. The frame is cropped and contrast-adjusted for illustrative purposes. The bead tracks inferred by our framework are overlaid on top of each other to assess consistency. **b**, Full confidence of bead 1’s x-track plotted as a shaded region in a time interval (10ms to 20ms); darker colors indicate higher confidence. **c**, The same layout as **b** but for bead 1’s y-track. **d**, Direct comparison of bead 1’s 1-bit *x*-track with its 4-bit and 8-bit *x*-tracks during the same time interval as in **b**. **e**, Direct comparison of bead 1’s 1-bit *y*-track with its 4-bit and 8-bit *y*-tracks during the same time interval as in **c**. **f**, Full confidence of all beads shifted together for comparison. Bead 1’s confidence is plotted in green, bead 2’s confidence is in red, and bead 3’s confidence is in blue. With this color scheme, white appears where the confidences overlap the most. **g**, The same layout as **f** but for the y-positions.

In the top right part of Fig. 2a, we plot the inferred particle tracks on top of the sum of all the binary frames. This dataset, which spans approximately 115ms, clearly shows three shifts of around 200nm in the particle positions. These shifts occur at approximately 15ms, 55ms, and 95ms, closely aligning with the expected dwell time of 40ms. Additionally, the directions of these shifts are consistent with the experimental settings: the first shift is to the left, the second is upward, and the third shift is downward.

In Fig. 2a, we align the three particle tracks by subtracting each track’s center of mass, enabling a direct comparison of their relative trajectories. Remarkably, the tracks exhibit sub-diffraction agreement—converging within approximately 50nm, despite being reconstructed from 1-bit-per-pixel data (5,750 frames with about five photons per bead per frame). We calculated the Euclidean distances between the aligned tracks to quantify this agreement. Across all frames, the maximum separation between any two particles is under 65nm, and the average pairwise distance is approximately 21nm—both well below the PSF radius of 250nm.

In addition, since the beads remain stationary during the dwell time, the spread of their inferred tracks during these periods provides a metric for quantifying tracking errors. Figure 2a shows that the beads remain fixed at the three vertices of the “L”-shaped trajectories, and the track spreads at these vertices are consistently below 50nm. This result demonstrates that our proposed framework achieves localization precision below the PSF width directly from 1-bit frames. We highlight that no method can currently achieve what is shown in this figure.

Furthermore, since fluorescent beads are known to remain stationary during dwell time, it is possible to sum the binary frames over time (binning) to perform conventional tracking. For this purpose, we binned 125 binary frames and used TrackMate^49^ to analyze the resulting frames. The tracks generated by TrackMate are also plotted in Figs. 2d and 2e, showing alignment, albeit at a lower spatial resolution, with the SP^2^T results. This strong correlation indicates that SP^2^T achieves a similar spatial resolution while offering a temporal resolution that is two orders of magnitude faster.

As for traditional (intensity-based) tracking tools, while selecting a smaller bin size for frames can improve the temporal resolution for TrackMate, this comes at the cost of reduced spatial resolution. As the bin size decreases, the limited information in each frame results in poorer localization accuracy, as quantified by the Cramér–Rao lower bound^42,50^.

Next, we compare the SP^2^T results with each other. As illustrated in Figs. 2a and 2b, even without assuming any correlation between the particle tracks, all three SP^2^T tracks align with each other within a range of 50nm after applying a constant translational shift. This precision is well below the diffraction limit of approximately 230nm in this dataset—calculated based on the system’s numerical aperture and emission wavelength—demonstrating the consistency and accuracy of SP^2^T in tracking particles.

### Aerolysin diffusing on a supported lipid bilayer

**Figure 3:**
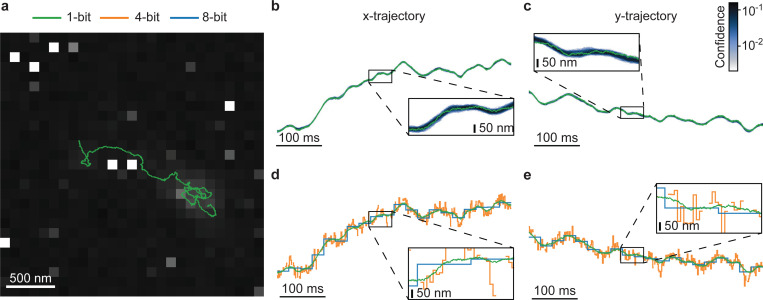
Results for Cy3B-labeled aerolysin diffusing on a SLB. **a**, Example frame obtained by summing all 5,100 individual 40×40 1-bit frames acquired at 100*μ*s exposure. The image has been contrast-adjusted for clarity, with the aerolysin track inferred by our framework overlaid. **b**, Confidence plot for the aerolysin’s x-coordinate trajectory, where darker shading denotes higher confidence. The boxed region is magnified for closer inspection. **c**, Same as **b**, but for the y-coordinate trajectory. **d**, As ground truth is unavailable in real experiments, we approximate a control by comparing the aerolysin’s 1-bit trajectory with 4-bit and 8-bit trajectories obtained using TrackMate^49^. For clarity, we zoom in on the same region shown in **b**. Note the discontinuity in the 4-bit trajectory, which arises from TrackMate’s failure to detect the particle under the low signal-to-noise ratio of the 4-bit frames. **e**, Same as **e**, but for the y-coordinate trajectory.

In the next example, we turn to pro-aerolysin monomers diffusing on a supported lipid bilayer (SLB). Aerolysin is a protein involved in Aeromonas-causing pathogenic infections^51^ and widely used in biosensing applications^52^. Although the exact mechanism of membrane association and pore formation has never been captured dynamically, in this case, we observe that most molecules appear immobile, suggesting strong interactions. However, a small fraction exhibits faster diffusion, indicating weaker membrane interactions. Unlike the fluorescent beads used in the previous dataset, these aerolysin monomers are labeled with Cy3B, yielding only about half the fluorescence brightness. Across 5,100 frames, this corresponds to about two detected photons on average per molecule per frame, making the tracking task substantially more challenging (see Fig. 3a).

This reduced brightness presents a robustness test to our framework. Despite this, as depicted in Figs. 3b and 3c, our framework achieves particle tracking with resolution below the diffraction limit—yielding localization precision on the order of 50nm using only 1-bit frames, consistent with the performance observed in the fluorescent bead dataset. In Figs. 3d and 3e, while we have no ground truth for the 1-bit track, we present a direct comparison between our tracks and those generated by TrackMate^49^. In the zoomed-in region, it is evident that the 4-bit tracks exhibit missing segments, attributed to the lower brightness of the aerolysin labels. This further highlights the limitations of the existing intensity-based paradigm when dealing with low-intensity signals and underscores the robustness of our approach.

### Short camera exposure for accurate diffusion analysis

**Figure 4:**
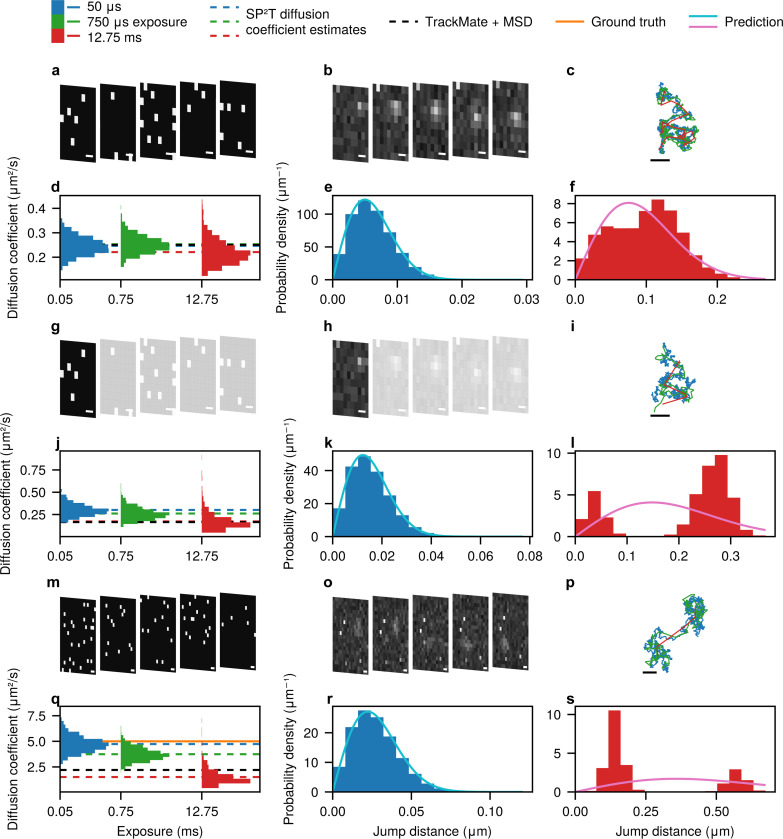
Effects of camera exposure on diffusion coefficient estimates, particle trajectories, and jump-distance distributions. All scale bars in this figure correspond to 200nm. **a**, Frames of a diffusing Atto565-labeled ganglioside GM1 acquired with the SPAD512^2^ camera (50*μ*s per frame; see Experimental methods). **b**, Effective 12.75ms-exposure frames of the same region, generated by summing consecutive short-exposure frames. **c**, Particle trajectories reconstructed by SP^2^T under different exposure conditions. **d**, Estimated diffusion coefficients across effective exposures. SP^2^T results are shown as blue, green, and red dashed lines; TrackMate^49^ results from 12.75ms frames are shown as a black dashed line. **e**, Jump-distance distribution between consecutive frames from native 50*μ*s data compared with the Rayleigh distribution (light blue) predicted from the mean diffusion coefficient estimated by SP^2^T (0.25*μ*m^2^/s). **f**, Same analysis as in **e**, but using the 12.75ms frames and the corresponding SP^2^T estimate (0.22*μ*m^2^/s); Rayleigh prediction shown in pink. **g–h**, Pseudo-stroboscopic dataset generated by retaining only the first frame of every five consecutive frames, reducing the effective frame rate while keeping exposure fixed. **i–l**, Analyses of the pseudo-stroboscopic dataset, arranged as in **c–f**. **m–s**, Same analyses applied to simulated data with diffusion coefficient 5*μ*m^2^/s (orange horizontal line in **q**). Simulation parameters match experiments: objective numerical aperture 1.49, pixel size 100nm, refractive index 1.52, 50*μ*s exposure, and emission rate of ∼15,000 photons/s (comparable to beads in Fig. 2).

Next, we highlight the critical importance of short camera exposures for accurately estimating diffusion coefficients and distinguishing between diffusive models in single-particle tracking. To demonstrate this, we use the SPAD512^2^ camera to acquire 1-bit image frames of diffusing Atto565-labeled ganglioside GM1 molecules with 50*μ*s per frame (see Fig. 4a). Gangliosides are glycosphingolipids that self-organize into dynamic, nanoscopic domains within biological membranes. The assembly of nanomains is driven predominantly by the hydrogen bonding capabilities of their sialic acid residues and the glycan headgroup^53–55^. Here, consecutive 1-bit frames are then summed to generate higher-bit-depth image stacks with effectively longer exposures. For example, summing 15 frames produces a 4-bit image with an effective exposure of 750*μ*s, while summing 255 frames yields an 8-bit image with an effective exposure of 12.75ms (see Fig. 4b). Subsequently, these datasets are analyzed with SP^2^T, which can handle images of any bit depth, to extract trajectories, estimate diffusion coefficients, and compute jump distance distributions.

As illustrated in Fig. 4c, shorter exposures (50*μ*s) produce trajectories with many more steps than those reconstructed from long-exposure frames, reflecting the much higher temporal resolution available when the same total number of 1-bit frames is used. This difference translates directly into diffusion coefficient estimates (Fig. 4d): for 50*μ*s exposures, the 95% credible interval (CI) spans 0.17*μ*m^2^/s to 0.32*μ*m^2^/s, whereas for 12.75ms exposures the interval broadens slightly to 0.14*μ*m^2^/s to 0.33*μ*m^2^/s. The increased uncertainty with longer exposures is expected, yet the absolute value we obtain is lower than the diffusion coefficient of (2.6 ± 1.3)*μ*m^2^/s reported previously in the literature^55^. We emphasize that, unlike these ensemble-averaged measurements over large fields of view, our estimate in Fig. 4d derives from a small region of interest containing a single GM1 molecule. Re-analyzing the same experimental sample with an EMCCD camera (512×512 pixels, 50ms exposure) using TrackPy (shown in Fig. S.3) reveals a wide distribution of diffusion coefficients, ranging from below 0.01*μ*m^2^/s to nearly 20*μ*m^2^/s, with about 18% of estimates falling between 0.1*μ*m^2^/s to 0.5*μ*m^2^/s—the range where our estimate lies. This breadth is consistent with the possibility of local inhomogeneities in the SLB^56,57^.

The impact of exposure time is even more pronounced in the jump-distance distribution (displacements between consecutive frames) and its interpretation. As shown in Fig. 4e, the distribution obtained from 50*μ*s exposures closely matches the Rayleigh distribution predicted from the mean diffusion coefficient estimated by SP^2^T using the same frames. Crucially, this Rayleigh curve is not a fit but a theoretical prediction, and the close agreement demonstrates that short exposures preserve the underlying diffusion dynamics with high fidelity. In contrast, the distribution derived from 12.75ms exposures deviates strongly from this prediction (Fig. 4f). This discrepancy suggests that long exposures can lead to spurious inference of the diffusive model; for example, the skewed distribution might be misinterpreted as evidence of heterogeneous diffusion or anomalous transport, when in fact it is an artifact of reduced temporal resolution.

To further investigate the impact of limited temporal resolution, we created a pseudo-experimental dataset by retaining only the first frame of every five consecutive frames, mimicking a stroboscopic illumination scheme^26–28^ (Figs. 4g and 4h). This reduces the effective frame rate by a factor of five while keeping the exposure per frame constant. Under this setup, trajectories reconstructed from 12.75ms exposures significantly underestimate the diffusion coefficient, yielding a mean of only 0.17*μ*m^2^/s (see Fig. 4j), consistent with recent observations^24^, and exhibit greater uncertainty (95% CI: 0.07*μ*m^2^/s to 0.41*μ*m^2^/s). Moreover, the jump-distance distribution (Fig. 4l) becomes bimodal. This bimodality arises as an artifact of having relatively few trajectory steps when exposure is prolonged. While such artifacts should diminish with sufficient statistics, the need for many more trajectories to recover the correct dynamics underscores the main conclusion: short exposures provide more reliable diffusion coefficient estimates and jump-distance distributions.

For comparison, Fig. S.4 presents results obtained when the other four frames in each five-frame block were retained instead of the first, while Fig. S.5 shows results from a different region of interest within the same dataset. Finally, we note that the same form of bias observed in Figs. 4j to 4l can also be reproduced *in silico* by simulating a fast-diffusing particle with diffusion coefficient of 5*μ*m^2^/s, without discarding frames, further emphasizing the need for adequate temporal sampling in single-particle tracking experiments.

These findings raise an important question: How short must the camera exposure be to reliably recover both the correct diffusion coefficient and the underlying diffusive model? Defining a strict threshold is difficult, as it depends on many factors, including the particle’s diffusion coefficient, fluorophore brightness, background noise, camera characteristics, and even the trajectory’s geometry. Nevertheless, our analysis in Fig. 4 reveals a clear trend: shorter exposures consistently produce more accurate and reliable results. Accordingly, we advocate minimizing exposure times wherever possible. This reinforces the importance of moving beyond the localization paradigm, which inherently relies on finite exposures to improve per-frame fits, and underscores the dual need for ultrafast imaging hardware such as SPAD arrays, together with tracking algorithms that can extract precise information from low-bit-depth, high-temporal-resolution data.

### Multiplexing

Having demonstrated the ability of SP^2^T to track real diffusing molecules, we now showcase its multiplexing capability by analyzing a dataset containing multiple diffusing aerolysin molecules across the full 512×512 pixel field of view—representing an over 100-fold increase in area compared to earlier examples—captured using the SPAD512^2^ camera from Pi Imaging. This dataset comprises 1,000 frames, with each molecule emitting, on average, approximately two photons per frame. Frames were captured with an exposure time of 100*μ*s, allowing us to evaluate SP^2^T’s performance under realistic, high-throughput imaging conditions.

**Figure 5:**
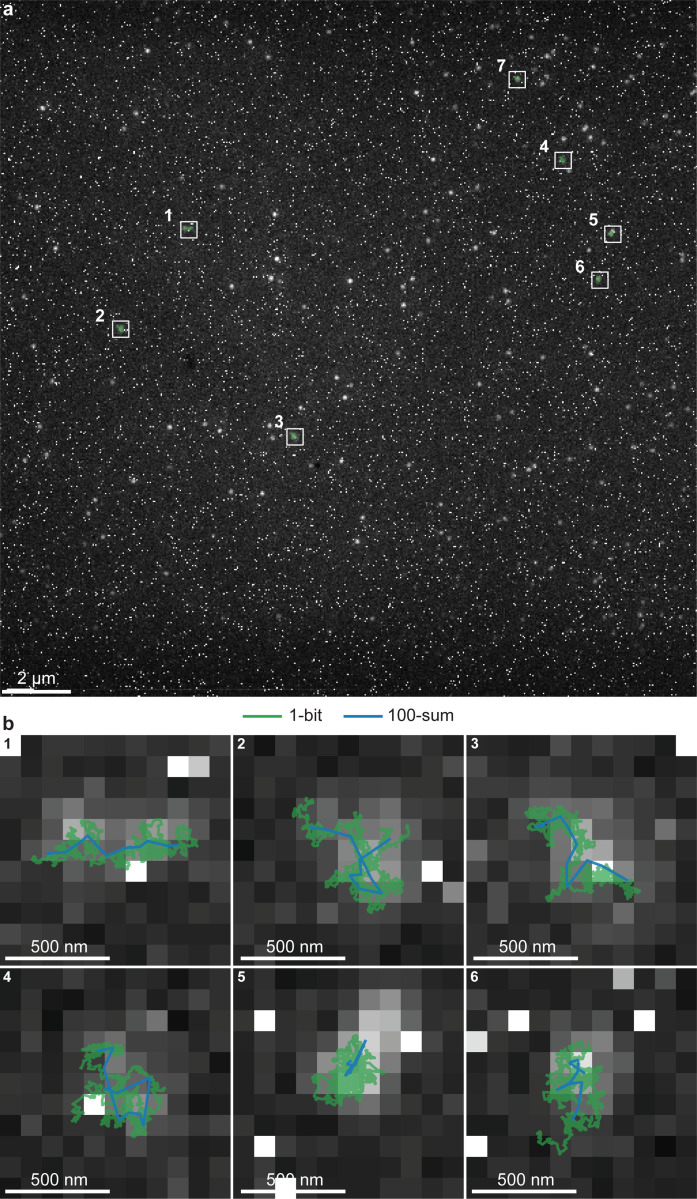
SP^2^T results for tracking diffusing aerolysin molecules across the full 512×512 pixel FOV of the SPAD512^2^ camera from Pi Imaging. The dataset consists of 1,000 frames with 100*μ*s exposure. **a**, A binned frame displaying the full FOV. Seven aerolysin molecules identified as nonstationary are highlighted with white boxes. Contrast is adjusted for visual clarity. **b**, Zoomed-in views of the selected regions containing six of these seven molecules. SP^2^T tracks are overlaid in green, while particle tracks extracted using TrackMate from 100-sum frames (10 sets of 100 consecutive frames summed) are shown in blue.

**Figure 6:**
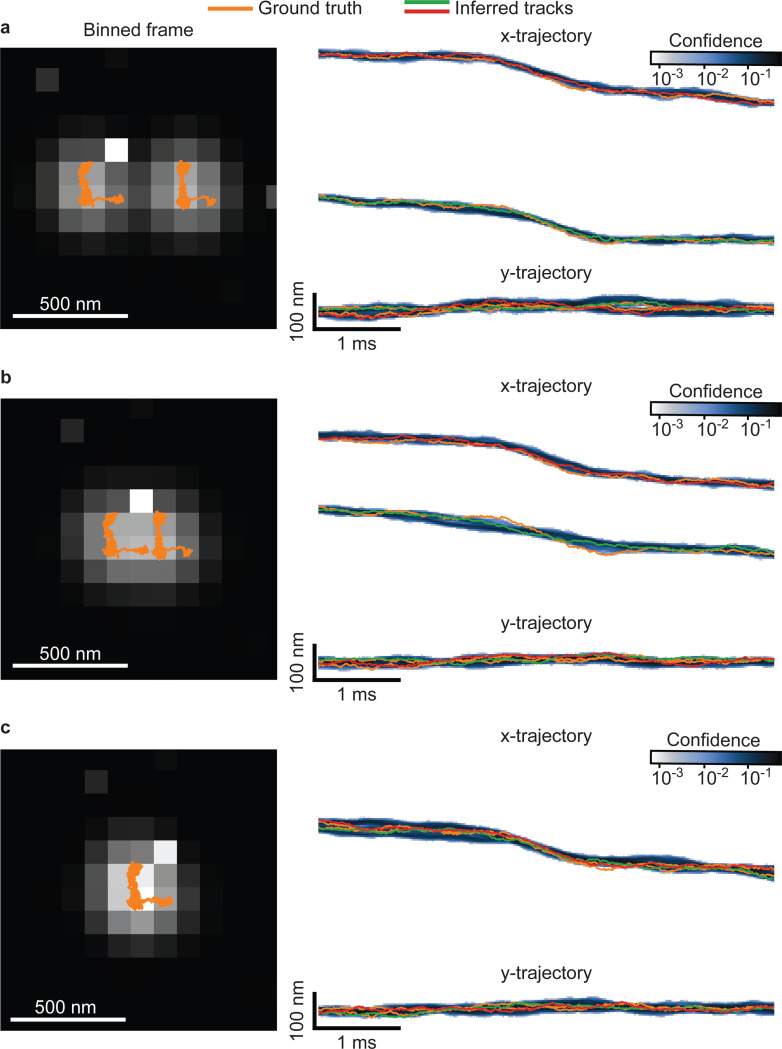
Performance of SP^2^T as particle trajectories are brought closer together. Simulated data were generated using the trajectories from Fig. 2, with a numerical aperture of 1.45, refractive index 1.515, emission wavelength 665nm, exposure time 10*μ*s, and background noise taken directly from Fig. 2. **a**, Summed 1-bit frames overlaid with ground-truth trajectories (orange) and MAP tracks inferred by SP^2^T (green and red). The average trajectory separation is 400nm; the right panel shows a zoomed-in region. Shading indicates tracking confidence, with darker colors corresponding to higher posterior probability of the emitter residing within a 10nm bin. **b**, Same as **a**, but with reduced spatial separation of 200nm. **c**, Same as **a**, but with complete spatial overlap (0nm).

Figure 5a presents the sum of all 1,000 frames for representation purposes only. Before processing this dataset, we first selected 5,000 frames (the 1,000 frames used for analysis, along with the subsequent 4,000 frames) and computed a temporal average to serve as a background calibration. This approach ensures that stationary particles are treated as part of the background and thus excluded from tracking, allowing SP^2^T to focus on dynamic molecules. Using this background calibration, SP^2^T identified seven diffusing aerolysin molecules. Six of these molecules were selected and plotted in Fig. 5b for optimal figure clarity only. We ascribe the stationarity of the bright spots (molecules) in this dataset, shown in Fig. 6a, to incomplete activation of the aerolysin molecules—specifically, the failure to cleave their C-terminal peptides^58,59^.

In Fig. 5b, we compare the 1-bit particle tracks inferred by SP^2^T with those extracted using TrackMate from 100-sum frames, where each frame is generated by summing 100 consecutive frames (10 sets in total). Consistent with our previous results, SP^2^T achieves particle tracking with a 100-fold improvement in temporal resolution while maintaining a spatial resolution comparable to that achieved with TrackMate on 100-sum frames.

### Robustness tests with synthetic data

In this section, we focus on using synthetic data to evaluate SP^2^T’s robustness in different parameter regimes (separation between emitting particles in Fig. 6, diffusion coefficient in Fig. S.6, brightness in Fig. S.7, and dark counts in Fig. S.8).

As illustrated in Fig. 6a, we used two estimated bead trajectories from Fig. 2 as ground truth, placing them side-by-side with a spatial separation (average displacement) of approximately 400nm. We then simulated 1-bit frames using real dark count calibrations (as in Fig. 2), fluorescence brightness levels consistent with Fig. 3, and the model described in Methods. In Fig. 6a, we present SP^2^T’s results in the same format as Figs. 2c, 2d, 3b and 3c. Consistent with previous findings, SP^2^T found the correct number of emitting particles, and the inferred tracks align with the ground truth trajectories within a range of around 50nm.

In the lower panels of Fig. 6, we gradually decreased the spatial separation between the two tracks in increments of 200nm, down to complete overlap (0nm separation), where the centers of the PSFs, each with a radius of approximately 250nm, coincide. As shown in Figs. 6b and 6c, SP^2^T maintained its accuracy even as the emitting particles overlapped almost entirely. This robustness is due to SP^2^T’s use of brightness calibration, allowing it to reliably infer the number of emitting particles even in challenging scenarios involving close proximity of the moving particles.

## Discussion

In this study, we demonstrated the robustness of SP^2^T, a tracking framework specifically designed to operate directly on binary frames from single-photon detectors. By removing the need for frame binning and conventional localization, SP^2^T delivers millisecond-to-microsecond improvements in temporal resolution while maintaining localization precision. The framework consistently recovered the correct number of particles and produced accurate trajectories across diverse datasets, ranging from controlled fluorescent bead experiments on a piezoelectric stage to biologically relevant cases involving Cy3B-labeled aerolysin monomers on SLBs and Atto565-labeled ganglioside GM1 molecules.

We have also compared SP^2^T to the widely used single-particle tracking tool, TrackMate^49^, across all datasets in this study. While TrackMate and other tracking tools^25,60,61^ rely on long-exposure frames to achieve high spatial resolution, they fundamentally rely on the notion of localization itself. In contrast, SP^2^T provides tracks with comparable spatial resolution by circumventing the localization paradigm while achieving over ten times the temporal resolution, eliminating blurring artefacts and enabling us to analyze both slowly diffusing organelles^47^ and faster-diffusing drug nanoparticles, as illustrated in Fig. 1b. This improvement holds promise in capturing faster dynamic processes, including vesicle transport and drug nanoparticle delivery, with a level of previously inaccessible temporal resolution.

Beyond the choice of tracking algorithm, our results help highlight a more fundamental issue: long camera exposures systematically bias the estimation of diffusion parameters and underlying diffusive models, especially for fast-moving particles. This challenge persists regardless of the tracking method used. To mitigate such biases, we advocate for the fastest possible exposure and SP^2^T, designed to analyze low-bit-depth data without requiring frame summation. Together, this hardware-software pairing delivers accurate, high-temporal-resolution tracking even in photon-starved conditions, significantly extending the experimental dynamic range for single-molecule studies.

Taken together, our findings point to the broader promise of SPAD arrays and related architectures capable of detecting low photon counts per pixel (and thus discrete intensities) for next-generation high-speed imaging. With various architectures tailored to diverse applications, SPAD-based detectors enable new experimental designs, but also demand rethinking traditional localization strategies. Analyzing single-photon data requires moving beyond frame-based processing paradigms relying on high photons and smooth intensities per pixel, and SP^2^T meets this need by tracking particles directly from photon arrival events. This shift enables more faithful recovery of molecular motion at the scales and speeds relevant to current biological investigations.

While our work has primarily focused on pixel-based SPDs, such as SPAD arrays, the framework can be adapted to non-array-based SPDs such as the LINCam^62^ and NCam^63–65^. These detectors provide an exciting opportunity to enhance spatial resolution even further by eliminating the need for spatial binning, just as we avoid temporal binning in our current approach. This adaptability might enable truly bin-free imaging in both spatial and temporal dimensions, optimizing resolution across all domains.

Additionally, without binning constraints, we could potentially distinguish between multiple molecular species by leveraging fluorescence lifetimes. Detectors like the LINCam^62^ and TCSPC-capable SPAD arrays^34^, coupled with pulsed laser sources, already capture lifetime information, enabling discrimination between different fluorophores based on their unique decay profiles^66^. This capability opens the door toward achieving higher resolution and multi-species tracking in complex environments, making SPDs a powerful platform for future imaging applications.

Although SP^2^T has shown significant advantages in tracking performance, there are areas of improvement. Specifically, because SP^2^T works directly with binary frames, the number of frames it analyzes is substantially higher—up to 255 times more—than that of a typical 8-bit grayscale image. This dramatic increase in the number of frames raises potential challenges regarding computational cost. Performing an analysis that jointly considers all binary frames could become computationally intensive for large datasets. With this in mind, we have implemented two primary strategies to address the computational challenges posed by the large number of binary frames. First, as detailed in the Methods section, the most computationally intensive task in SP^2^T—likelihood calculation—is parallelized in a two-layer structure and executed on a GPU. This parallelization significantly accelerates processing, achieving up to a 50-fold speedup on a GTX 1060 GPU compared to a conventional CPU setup like the i7–7700k.

The second approach to reducing SP^2^T’s computational demands is to offer users the flexibility to operate in either parametric or nonparametric mode. In parametric mode, users specify the number of particles, allowing SP^2^T to bypass the computationally intensive process of particle count estimation. Users may initially run in nonparametric mode, then switch to parametric mode for a five-fold performance boost. This adaptability enables users to tailor the analysis to their needs, balancing computational efficiency with the desired analytical depth for their application. Furthermore, we recommend using tracks obtained from long-exposure frames as input to SP^2^T, which can yield a further twofold speed increase.

With these computational optimizations, SP^2^T can process over 5,000 50×50 frames in just a couple of hours using an NVIDIA GTX 1060. Additionally, its computational cost remains stable, regardless of the number of frames or particles, until the GPU cores reach saturation.

Owing to their versatility, SPDs are increasingly replacing conventional integrative detectors in diverse applications, including fluorescence lifetime microscopy^31,35^, image-scanning microscopy^32,34^, and Förster resonance energy transfer^35^. In a similar spirit, SP^2^T, as a mathematical framework built to operate directly on single-photon data, can be adapted to a variety of experimental configurations. For example: (1) SP^2^T can be extended to 3D tracking or specialized PSFs by modifying the integrand in equation (4) in the Methods, enabling direct inference in three dimensions; and (2) for SPDs capable of recording photon arrival times, SP^2^T could couple equation (5) with an arrival-time distribution, allowing simultaneous tracking and species discrimination based on fluorescence lifetimes.

As an emerging technology, SPDs and computational methods designed to exploit their capabilities fully are still in their early stages, with significant untapped potential. Continued advances in hardware design, algorithmic innovation, and software optimization will further enhance their performance and accessibility. Given these ongoing developments, SPDs are poised to transform biological imaging by enabling investigations at previously inaccessible spatiotemporal scales, opening new frontiers in our understanding of dynamic biological events.

## Methods

### Theoretical framework

This section outlines the mathematical framework of SP^2^T. For notational simplicity, we first define the following terms: N represents the number of frames, P denotes the number of pixels per frame, $w_{n}^{p}$ refers to the data collected at pixel p on frame n, M indicates the number of particles, $x_{n}^{m}$ represents the m-th particle’s position at frame n, and MSD is the mean squared displacement, as later defined in equation (10). With these definitions, we can express all collected data as $w_{1:N}^{1:P}=\left\{w_{n}^{p}|n=1,\ldots ,N,p=1,\ldots ,P\right\}$. Similarly, the collection of all particle tracks is denoted as $x_{1:N}^{1:M}$.

As discussed in the sections above, the goal of SP^2^T is to determine all variables of interest (including the number of light emitting particles, particle tracks, and the MSD) along with the corresponding credible intervals. We achieve this by constructing the full probability distribution, known as the posterior, $\mathbb{P}\left(M,x_{1:N}^{1:M},\;\text{MSD}|w_{1:N}^{1:P}\right)$, representing the probability distribution of all variables of interest given the collected data. Following Bayes’ theorem, this posterior can be expressed as

(1) $$\mathbb{P}\left(M,x_{1:N}^{1:M},\;\mathrm{MSD}|w_{1:N}^{1:P}\right)=\frac{\mathbb{P}\left(w_{1:N}^{1:P}|M,x_{1:N}^{1:M},\;\text{MSD}\right)\mathbb{P}\left(M,x_{1:N}^{1:M},\;\text{MSD}\right)}{\mathbb{P}\left(w_{1:N}^{1:P}\right)}.$$

The first term in the numerator, $\mathbb{P}\left(w_{1:N}^{1:P}|M,x_{1:N}^{1:M},\;\text{MSD}\right)$, represents the likelihood reflecting the probability distribution of the data given the variables of interest. This term usually describes the system’s physics (the model for photon detection in this context) and realistic complications such as hot pixels. The second term, $\mathbb{P}\left(M,x_{1:N}^{1:M},\;\text{MSD}\right)$, known as the prior, imposes additional constraints on the variables of interest, such as a particle’s motion model and ensuring the MSD remains non-negative. The denominator, $\mathbb{P}\left(w_{1:N}^{1:P}\right)$, is referred to as the evidence and is generally treated as a normalization constant, as it does not depend on any variable of interest.

In the following sections, we will establish the base likelihood using only the photon detection model. Next, we will generalize this base likelihood to account for the aforementioned realistic complications and then discuss the prior we place on the variables of interest. Finally, we will briefly introduce our approach to constructing and sampling the posterior.

#### Base likelihood

As introduced above, the likelihood $\mathbb{P}\left(w_{1:N}^{1:P}|M,x_{1:N}^{1:M},\;\text{MSD}\right)$ represents the probability distribution of the data given the number of particles, the particle tracks, and the MSD. To express this likelihood mathematically, we first observe that once all particle tracks are known, neither the number of particles nor the MSD affects photon detection. This observation allows us to simplify $\mathbb{P}\left(w_{1:N}^{1:P}|M,x_{1:N}^{1:M},\;\text{MSD}\right)$ to $\mathbb{P}\left(w_{1:N}^{1:P}|x_{1:N}^{1:M}\right)$.

Furthermore, considering that we are dealing with fluorescently emitting particles whose lifetimes (on the order of nanoseconds^67^) are significantly shorter than the frame period (at least 10*μ*s), the probability that an emitting particle excited during the exposure period of one frame will result in a photon detection in the next frame is negligible (less than 10^−200^, assuming a 10ns lifetime and excitation occurring in the middle of an exposure). Therefore, it is reasonable to assume that particle positions at frame n do not influence other frames. This assumption allows us to further simplify the likelihood as a product

(2) $$\mathbb{P}\left(w_{1:N}^{1:P}|x_{1:N}^{1:M}\right)=\prod_{n=1}^{N}\mathbb{P}\left(w_{n}^{1:P}|x_{n}^{1:M}\right).$$

Similarly, since the pixels of a detector usually operate independently, we can express the per-frame likelihood $\mathbb{P}\left(w_{n}^{1:P}|x_{n}^{1:M}\right)$ as a product over the individual pixel likelihoods

(3) $$\mathbb{P}\left(w_{n}^{1:P}|x_{n}^{1:M}\right)=\prod_{p=1}^{P}\mathbb{P}\left(w_{n}^{p}|x_{n}^{1:M}\right).$$

The per-pixel likelihood $\mathbb{P}\left(w_{n}^{p}|x_{n}^{1:M}\right)$ is influenced by four key factors: the PSF of the optical system, the pixel area A, the brightness of the particles h, and the number of particles M. The PSF characterizes how light from a point source is distributed across the detector^68^. At the same time, brightness represents the expected number of photons arriving at a pixel perfectly aligned with the PSF of a particle located at the focal plane. The total number of photons received by pixel p from all emitting particles, denoted as $u_{n}^{p}$, can be expressed as

(4) $$u_{n}^{p}=h\sum_{m=1}^{M}\iint_{A}\mathrm{PSF}\left(x,y;x_{n}^{m}\right)\;\text{d}x\;\text{d}y.$$

Since a single-photon detector can only distinguish between the absence of photons (false) and the presence of at least one photon (true), the measurement $w_{n}^{p}$ must be sampled from a Bernoulli distribution. The parameter of this Bernoulli distribution is determined by the probability of detecting at least one photon, which is given by $1-\text{Poisson}\left(0;u_{n}^{p}\right)$. Here, $\text{Poisson}\left(0;u_{n}^{p}\right)=\exp\left(-u_{n}^{p}\right)$ represents the probability of detecting zero photons. Consequently, the probability of detecting at least one photon is $1-\exp\left(-u_{n}^{p}\right)$, and the corresponding mathematical expression is

(5) $$w_{n}^{p}\sim \mathrm{Bernoulli}\left(1-\exp\left(-u_{n}^{p}\right)\right).$$

Thus, the overall likelihood can be obtained by combining equations (2) to (5).

#### Photophysical states

A crucial factor to consider is the photophysical state of a particle: whether it is in a bright state, actively emitting photons, or in a dark state, where no emission occurs. We define the photophysical state of particle m at frame n as a binary variable $f_{n}^{m}$, where $f_{n}^{m}=1$ indicates an emitting particle and $f_{n}^{m}=0$ represents a non-emitting state. Currently, SP^2^T takes $f_{1:N}^{1:M}$ as an input and treats these states as fixed throughout the analysis. Incorporating $f_{n}^{m}$ into equation (4) modifies the expected intensity $u_{n}^{p}$ at pixel p in frame n as follows

(6) $$u_{n}^{p}=h\sum_{m=1}^{M}f_{n}^{m}\iint_{A}\mathrm{PSF}\left(q_{x},q_{y};x_{n}^{m}\right)\;\text{d}q_{x}\;\text{d}q_{y}.$$

Here, $q_{x}$ and $q_{y}$ are dummy variables used to perform the integration over the pixel area.

#### Dark counts

Single-photon detectors are known to exhibit dark counts^34^, which are false detections of photons that occur even in the absence of light. These dark counts, specific to each pixel, arise due to thermal noise and other intrinsic factors within the detector. As a result, the likelihood model must be adjusted to account for these dark counts, which contribute to an additional noise source and affect the accuracy of photon detection. To do so, we introduce a quantity that represents the expected number of dark counts per frame exposure, ${\text{DC}}^{p}$, and we modify equation (6) as follows

(7) $$u_{n}^{p}={\text{DC}}^{p}+h\sum_{m=1}^{M}f_{n}^{m}\iint_{A}\mathrm{PSF}\left(q_{x},q_{y};x_{n}^{m}\right)\text{d}q_{x}\;\text{d}q_{y}.$$

#### Other pixel defects

Similar to dark counts, pixels of a single-photon detector can also carry other defects. For example, some pixels may be imperfectly connected to the circuit and, therefore, not produce any signal during an experiment. We, therefore, allow users to set a Boolean label, $F^{p}$, which is zero when the corresponding pixel has a defect and one otherwise, to determine whether a pixel should be excluded from the likelihood calculation. Specifically, we change equation (3) to

(8) $$\mathbb{P}\left(w_{n}^{1:P}|x_{n}^{1:M}\right)=\prod_{p=1}^{P}{\left[\mathbb{P}\left(w_{n}^{p}|x_{n}^{1:M}\right)\right]}^{F^{p}}.$$

#### Full likelihood

To summarize the sections above, we present the mathematical form of the full likelihood here:

(9) $$\mathbb{P}\left(w_{1:N}^{1:P}|x_{1:N}^{1:M}\right)=\prod_{n=1}^{N}\prod_{p=1}^{P}{\left[\mathrm{Bernoulli}\left(w_{n}^{p};1-\exp\left(-u_{n}^{p}\right)\right)\right]}^{F^{p}},$$

where the expected photon count $u_{n}^{p}$ is given by equation (7).

#### Motion model prior

Now that we have established the full likelihood $\mathbb{P}\left(w_{1:N}^{1:P}|x_{1:N}^{1:M}\right)$, we briefly introduce the motion model prior so that the full posterior can be constructed. As mentioned earlier, we characterize the motion of a particle using the MSD. Specifically, we choose

(10) $$x_{n}^{m}\sim {\mathrm{Normal}}_{3}\left(x_{n-1}^{m},\;\text{MSD}\right).$$

#### Posterior

According to equation (1), we can now write the full posterior as

(11) $$\begin{array}{l} \mathbb{P}\left(M,x_{1:N}^{1:M},\;\mathrm{MSD}|w_{1:N}^{1:P}\right)\\ \propto \left[\prod_{n=1}^{N}\prod_{p=1}^{P}{\left[\mathrm{Bernoulli}\left(w_{n}^{p};1-\exp\left(-u_{n}^{p}\right)\right)\right]}^{F^{p}}\right]\left[\prod_{n=2}^{N}{\mathrm{Normal}}_{3}\left(x_{n}^{m};x_{n-1}^{m},2\text{MSD}\right)\right]\\ \times \mathbb{P}\left(x_{1}^{1:M}\right)\mathbb{P}(M)\mathbb{P}(\text{MSD}). \end{array}$$

Here, the last three priors $\mathbb{P}\left(x_{1}^{1:M}\right)$, ℙ(M), and ℙ(MSD) are chosen for computational convenience. As they primarily define the support of each variable and do not otherwise integrate critical physics, we introduce them in the Supplementary Information.

### Parallelization

Here, we parallelize: 1) the likelihood calculation at each frame; and 2) within each frame. Conceptually, this parallelization scheme stems from the notion that the full likelihood can be expressed as the product of per-pixel likelihoods across all pixels and frames. As an important technical point, these per-pixel likelihoods are independent when conditioned on particle tracks $x_{1:N}^{1:M}$ and per-pixel boolean label $F^{p}$ as shown in equations (8) and (9) so long as SPD pixels are uncorrelated. This independence allows us to parallelize calculations efficiently. Inspired by parallelization schemes across Bayesian applications^69–72^, we specifically tailor our framework to SPDs to achieve the best possible computational efficiency.

### Experimental methods

#### SPAD array

Experiments were performed on a custom-built widefield single-molecule fluorescence microscope equipped with a time-gated large 512×512 SPD array, SPAD512^2^ camera from Pi-Imaging with each pixel capable of detecting single photons, described previously^73^. This commercial version of the SwissSPAD^2^ camera was developed by the AQUA group (of Prof. Charbon) at EPFL. The camera captures binary images at an exceptional frame rate of up to 97,700 frames per second. A supercontinuum pulsed laser source was used for illumination, which could output an arbitrary wavelength in the visible range. We used either epi-illumination or total internal reflection illumination, allowing monitoring thousands of individual molecules simultaneously within a ≈50 × 50*μ*m^2^ FOV. The detection path can be swapped for instrument comparison with a conventional EMCCD camera.

#### Beads and piezo stage

Widefield imaging for the piezo dataset was performed using another custom fluorescence microscope, as described in Ronceray *et al.*^74^. Briefly, we imaged fluorescent beads while following a programmed trajectory set by the motion of the sample placed on a piezoelectric stage (Mad City Labs).

#### Material

We purchased 1,2-dioleoyl-sn-glycero-3-phosphocholine (18:1Δ9-CisPC, DOPC) dissolved in chloroform, Cholesterol and Brain SM Sphingomyelin from Avanti Polar Lipids, HEPES from Chemie Brunschwig AG, and glucose and GM1 ganglioside from Sigma-Aldrich.

#### Aerolysin Sample Preparation

Full-length wild-type aerolysin protein in the pET22b vector with a C-terminal hexa-histidine tag was used. Cysteines were introduced into specific pore regions by mutating amino acids like G68, enabling labeling and tracking conformational changes during pore formation^59^. The mutants were generated by Genscript using the wild-type plasmid as a template, as was previously done for the production of 26 other aerolysin mutants^75^. Here we followed protocols for aerolysin production, purification, labeling, and preparation of supported lipid bilayers already established in the Radenovic lab^73,75^.

#### Ganglioside Samples Preparation

For these measurements, we focused on the diffusing GM1 (monosialotetrahexosylganglioside, a glycosphingolipid with a single sialic acid residue known for its essential role in membrane organization and cell signaling) within DOPC-supported lipid bilayers. Supported lipid bilayers were prepared using 1,2-dioleoyl-sn-glycero-3-phosphocholine (DOPC)/cholesterol/sphingomyelin/GM1 (61:25:10:4, wt%) with 0.5% Allyl-PEG5000, and GM1–Atto565 was incorporated at a lipid-to-dye ratio of 1:15,000,000 to optimize imaging conditions. GM1 was a kind gift from Dr. Ilya Mikhaylov, whose synthesis was described in Svobodová *et al.*^55^. Lipid films were generated by evaporating chloroform-dissolved components under vacuum, rehydrated in buffer (10 mM HEPES, 10 mM NaCl, 170 mM glucose, pH 7.4, 1 mM total lipid), and vortexed thoroughly. SUVs were obtained by 90 min sonication and separated by centrifugation at 16,500 × g for 20 min. To form supported bilayers, 60 *μ*L of SUVs, 140 *μ*L buffer, and 0.6 *μ*L 1 M CaCl_2_ were incubated in coverslip chambers for 90 min, followed by extensive washing (20 × 100 *μ*L buffer) to remove excess material.

## Supplementary Material

Supplement 1

## Data Availability

We provide all the data analyzed in this paper as part of the Supplementary Information.

## Code availability

Our code is publicly available at https://github.com/LabPresse/SP2T.jl.
